# From ideal to real: a qualitative study of the implementation of in situ interprofessional simulation-based education

**DOI:** 10.1186/s12909-022-03370-2

**Published:** 2022-04-21

**Authors:** Mindy Ju, Naike Bochatay, Kathryn Robertson, James Frank, Bridget O’Brien, Sandrijn van Schaik

**Affiliations:** 1 Department of Pediatrics, University of California, San Francisco, Box 0106, 550th 16th Street 5th floor, CA 94158 San Francisco, USA; 2 Department of Pediatrics, Kaiser Permanente Santa Clara, 700 Lawrence Expressway, CA 95051 Santa Clara, USA; 3 Department of Medicine, University of California San Francisco, Clement St, CA 4150, 94121 San Francisco, USA; 4 Department of Medicine, University of California San Francisco, Box 0710, 533 Parnassus Ave, Floor 02, Room 2230, CA 94143 San Francisco, USA

**Keywords:** Continuing education, Interprofessional collaboration, Interprofessional simulation, Simulation, Teamwork

## Abstract

**Background:**

Despite the widespread adoption of interprofessional simulation-based education (IPSE) in healthcare as a means to optimize interprofessional teamwork, data suggest that IPSE may not achieve these intended goals due to a gap between the ideals and the realities of implementation.

**Methods:**

We conducted a qualitative case study that used the framework method to understand what and how core principles from guidelines for interprofessional education (IPE) and simulation-based education (SBE) were implemented in existing in situ IPSE programs. We observed simulation sessions and interviewed facilitators and directors at seven programs.

**Results:**

We found considerable variability in how IPSE programs apply and implement core principles derived from IPE and SBE guidelines with some principles applied by most programs (e.g., “active learning”, “psychological safety”, “feedback during debriefing”) and others rarely applied (e.g., “interprofessional competency-based assessment”, “repeated and distributed practice”). Through interviews we identified that buy-in, resources, lack of outcome measures, and power discrepancies influenced the extent to which principles were applied.

**Conclusions:**

To achieve IPSE’s intended goals of optimizing interprofessional teamwork, programs should transition from designing for the ideal of IPSE to realities of IPSE implementation.

**Supplementary information:**

The online version contains supplementary material available at 10.1186/s12909-022-03370-2.

## Background

Interprofessional Simulation Based education (IPSE), is a form of interprofessional education (IPE), where students from two or more professions learn with, from and about each other, during simulated patient care scenarios [1]. IPSE is an educational strategy with potential to enhance interprofessional collaboration, optimize teamwork and ultimately improve patient care [2–4]. Despite widespread adoption of IPSE, there is evidence that poor interprofessional collaboration (IPC) and communication continues to hamper the function of interprofessional teams, leading to compromised safety and quality of patient care [2–4]. Thus, the effectiveness of IPSE in reaching its stated goals can be questioned.

Studies of IPE and simulation-based education (SBE) offer some insight into potential shortcomings of IPSE, largely related to implementation. A rich literature describes the challenges associated with IPE, including professional silos, power differentials and hierarchy [5–8]. SBE comes with its own set of implementation challenges, which explains why the benefits of SBE described in the controlled setting of educational research may not be seen when put into practice [9, 10]. Furthermore, IPSE is often delivered as in situ SBE, meaning that it takes place in the actual clinical environment with participants who are members of the team that works in that environments in real life. In situ simulation has many benefits: it can assist in detecting latent hazards, facilitates attendance due to the workplace setting, and can increase organizational learning compared to other simulation settings [11]. Yet, in situ simulation comes with increased resource and time demands on the simulation team, technical challenges related to equipment, need to coordinate space, privacy issues and concerns about patient perception [12].

Taken together, IPSE is a highly complex modality requiring significant resources, yet little is known about the actual implementation of IPSE programs. As a first step to creating more effective IPSE, we conducted the current study to examine features of in situ IPSE programs across multiple disciplines and institutions. We created a framework based on existing guidelines for IPE and SBE to explore the extent to which programs abided by such guidelines and what challenges exist. Our findings can provide insight into factors that may contribute to the implementation success of IPSE, and inform strategies to improve IPSE in ways that can help achieve the goals of improved teamwork and patient care.

## Methods

### Design

We used case study methodology [13] to examine seven in situ IPSE programs in Northern California. We based our analysis on the framework method [14] - a form of thematic analysis, originating in social policy research. The defining feature of this method is the “matrix”, which is composed of rows (cases) and columns (codes). This structure enables analysis by both case and code. For our work, IPSE programs represent cases, while each code represents an IPSE principle from the literature.

### Cases: seven IPSE programs

We included seven IPSE programs as cases, using the following purposive sampling strategy. First, through the University of California San Francisco (UCSF) Kanbar Center for Simulation contact list, we identified eight in situ IPSE programs in Northern California and reached out to leaders from these programs to inquire whether they defined their programs as “interprofessional”. We then observed one session in each program to verify the interprofessional nature, after which we excluded one program because only one profession participated. The final seven programs represented different contexts. They included five hospitals (the UCSF Benioff Children’s Hospital, UCSF Medical Center, Zuckerberg San Francisco General Hospital, San Francisco Veterans Affairs Healthcare System, and the University of California, Davis) across five specialties (anesthesia, emergency medicine, internal medicine, obstetrics and gynecology, and pediatrics). These programs varied in number of professions involved, participants’ level of training, acuity of scenarios, and profession(s) within which the IPSE program resides (Table 1). We assigned a letter to each of the seven programs to preserve their anonymity (A-G).

**Table 1:** Description of seven interprofessional simulation-based education programs

	A	B	C	D	E	F	G
**Facilitators**	2 MDs, 2 RNs	2 RNs, 1 MDs	2–3 midwives	2 MDs	1 MD, 1–2 RNs	1 RN, 1 MD, simulation fellows	2 MDs, 1–2 simulation fellows
**Participants (N)**	~ 10	6–10	6–10	10–15	10–15	15–25	15–25
**Participant professions**	MDs, RNs, pharmacy	Mostly RNs, 1–2 MDs, pharmacy	Midwives, RNs, MDs	Mostly MDs, 1–2 RNs, 1 pharmacy	MDs, RNs	MDs, RNs, pharmacy	MDs, RNs, pharmacy
**Frequency of occurrence**	Twice a month	4 times a year	Once a month	Twice a month	Once a month	Once a week	Twice a month
**Length of a session (minutes)**	60 for MDs and pharmacists, 120 for RNs	120 min	90 min	60 min	60 min	60 min	60 min
**Number of scenarios per session**	2	2	1–2	1	1	1	1
**Use of mannequin**	Yes	Yes	No (standardized patient)	Yes	Yes	Yes	Yes
**Location of simulation session**	Dedicated room on the floor	Dedicated room on the floor	Variable but on the wards	Dedicated room on the floor	Dedicated room in the ED	On the wards	On the wards
**Program start year**	2006	2010	~ 2010	~ 2016	2017	2007	2015

### Codes: 12 principles of interprofessional simulation-based education

 Three investigators (MJ, BO’B, SVS) identified frequently cited evidence-based guidelines. These included guidelines for IPE developed by the Centre for the Advancement of Interprofessional Education in the UK [15] and the World Health Organization [16], as well as guidelines for SBE [17–19] and IPSE [20], which were developed by experts in the field. From these five publications we distilled “guiding principles” to create the 12 codes for our framework (Table 2), which helped structure our observation and interviews. All investigators reviewed the codes for their relevance to IPSE and met to discuss and reconcile differences in opinion.

**Table 2:** Principles of interprofessional simulation-based education and data collection methods for each principle

	Principle	Description	Source	Observations	Interviews
1	Equitable Distribution	Planning, implementation and learning are done jointly at every level involving all relevant professions. Distribution of roles and responsibilities across professions is balanced.	WHO IPE/CAIPE IPE/Boet IPSE	x	x
2	Active Learning	Activities that promote learners’ cognitive engagement (active participants, not passive bystanders) using strategies such as multiple repetitions, feedback, task variation, or intentional task sequencing	BEME SBE/Cook SBE	x	x
3	Interprofessional Competency-Based Learning Objectives	Program have clearly defined learning objectives that focus on competencies for collaborative practice, including interprofessional knowledge, behaviors/skills, and attitudes.	Boet IPSE/WHO IPE/CAIPE IPE/BEME SBE	x	x
4	Interprofessional Competency-Based Assessment	Program assess learners’ achievement of clearly defined outcomes or benchmarks for competency in collaborative practice, including interprofessional knowledge, behaviors/skills, and attitudes.	Boet IPSE/WHO IPE/CAIPE IPE/BEME SBE	x	x
5	Psychological Safety	The program sets up an atmosphere of social acceptance of feedback from all peers, notably through the physical environment and through pre-briefing.	BEME SBE/WHO IPE/Boet IPSE	x	x
6	Repeated and Distributed Practice	There is an opportunity for learners to engage in focused, repeated practice where the intent is skill improvement over a period of time.	Cook SBE/BEME SBE	x	x
7	Attention to Differences and Hierarchy	Educators discern and address diversity and differences between groups in educational, professional, and cultural background with sensitivity. They also raise issues related to power inequities between learners.	CAIPE IPE/Boet IPSE	x	x
8	Feedback during Debriefing	Debriefing occurs during the simulation session and includes feedback and information on performance provided to learners. Feedback can be provided by facilitators and by peers. Debriefing should be attributed to most experienced educators.	BEME SBE/Cook SBE/Boet IPSE	x	x
9	Sociological Fidelity	Scenarios have high levels of social realism and reflect how teams in real life are arranged.	BEME SBE/WHO IPE/Boet IPSE	x	x
10	Program Evaluation	Programs are rigorously evaluated as early as possible and involve major stakeholders. The purpose of the evaluation is stated clearly and considers learning outcomes and theoretical perspectives. Results from program evaluation may be disseminated.	CAIPE IPE/WHO IPE/Boet IPSE		x
11	Train Facilitators	Educators receive training to understand the ethos, principles and methods for IPSE. This training focuses on how to develop, deliver, and evaluate interprofessional simulation-based education.	CAIPE IPE/WHO IPE/Boet IPSE		x
12	Institutional Support	Institutional policies support educators and health workers to promote IPSE. They provide adequate financial support (remuneration models, funding streams, incentives for workers to participate), adequate time allocations (regular meetings for interprofessional champions), and adequate space and facilities.	WHO IPE/CAIPE IPE/Boet IPSE		x

### Data collection

Four investigators (NB, JF, MJ and BO’B) performed direct observations of IPSE sessions. At least two investigators observed each program to ensure diversity of viewpoints and to account for different degrees of familiarity with the setting. Three investigators (NB, MJ and KR) conducted and audio-recorded semi-structured interviews with IPSE session facilitators and program developers. The UCSF and UC Davis Institutional Review Boards reviewed the study and determined it to be exempt. All methods were performed in accordance with the relevant institutional ethical guidelines and regulations for exempt research, including the requirement for verbal versus written consent. Per UCSF and UC Davis Institutional Review Boards’ policy on exempt research, researchers obtained verbal consent from all participants at the beginning of each interview.

### Instruments

#### Observation guide

We created an observation guide based on the 12 IPSE principles we synthesized from the literature. We anticipated that three of the 12 principles would not be observable during the simulation sessions: “program evaluation”, “train facilitators”, and “institutional support”. We therefore omitted these from the observation guide and relied on interview data to obtain information about these three principles. One investigator (MJ) pilot tested during the first 3 observations and met with one other investigator (B’OB) to review the experience and the notes that were taken, leading to modifications to increase ease of use and include space for reflexivity on the observation guide.

#### Interview guide

After we completed all observations, we developed a semi-structured interview guide (Additional file 1). The interview guide addressed each of the 12 principles and aimed to elicit interviewees’ perspectives on affordances and barriers to applying the principles. We reviewed data from observations to inform the interviews but did not directly discuss these data with interviewees. We feared that doing so would create a sense of blame and failure in interviewees if their program did not implement each of the principles. We piloted the interview guide with a faculty member familiar with in situ simulation but not directly connected to any of the programs in our study. All interviews were professionally transcribed and identifying information was removed.

### Data analysis

We imported all observations and interview transcripts into qualitative data analysis software (Dedoose, SocioCultural Research Consultants, Manhattan Beach, CA, USA) to analyze the data. Transcribed data from all sources were de-identified. Three investigators (NB, MJ and KR) coded the data in an iterative manner. They coded each transcript independently and regularly met to reconcile any differences in opinion. We then used the framework to organize the data by cases (IPSE programs) and codes (IPSE principles) in a matrix. We collated excerpts from each source of data (observations and interviews) for a given program and given principle. Two authors (NB and MJ) separately reviewed these collated excerpts and assigned a designation of fully present, partially present and absent. We then met to reconcile differences in our designation. While we primarily used a deductive approach utilizing the predetermined list of codes (the 12 principles), we remained open to other insights gathered from our observations and interviews to ensure we captured all elements and perceptions of IPSE. In particular, we developed more codes to capture affordances and barriers to applying the principles during simulation sessions or as part of the programs.

### Investigator characteristics and reflexivity

The investigator team consisted of two medical education qualitative researchers (BO’B, NB) and four physician educational researchers from critical care clinical backgrounds (MJ, KR, SVS and JF). The four physician investigators also functioned as facilitators in two of the IPSE programs selected as cases for this study. We ensured that members of the investigator team who were not directly involved with these IPSE programs conducted the observations and interviews.

 As part of the observation guide, investigators reflected on their presence as an observer, including any interactions with participants and feelings experienced while observing.

## Results

Between August 2016 and August 2017, we observed three simulation sessions for each program, for a total of 21 sessions (30 h). Between March 2019 and September 2019, we interviewed two facilitators and/or program developers from each program. At Program E, we interviewed one program developer because this program shut down in the period between observations and interviews. Altogether, we interviewed 13 program developers and facilitators. All programs were held in situ and structured the simulations around patient care scenarios that constituted clinical emergencies with an aim to allow team members to practice patient management and interprofessional teamwork in the context of the simulation setting.

### Application of principles

Table 3 summarizes our framework matrix, demonstrating the application of IPSE principles (codes) across IPSE programs (cases). We found that all 12 principles we identified were applicable in the context of IPSE and were endorsed by interviewees. However, we noted considerable variation in the application of the 12 principles across the seven programs, with some principles applied by most programs (e.g., “active learning”, “psychological safety”, “feedback during debriefing”), whereas others were rarely applied (e.g., “interprofessional competency-based assessment”, “repeated and distributed practice”). We also noted that some programs applied most principles (programs A, B, C, E), whereas other programs applied fewer principles (programs D, F, G). None of the programs fully applied all principles. Instead, they often applied principles in a partial way, meaning that they consistently applied principles but not to their full extent. For example, “institutional support” was partially applied: all programs were recognized by their institution and participants’ attendance was encouraged but the programs often lacked sufficient resources. As another example, “equitable distribution” was in many programs exemplified by learning across professions, but few had interprofessional representation in the planning or implementation process.

**Table 3:** Application matrix of principles of interprofessional simulation-based education at seven programs

Principle	Equitable Distribution	Active Learning	Interprofessional Competency-Based Learning Objectives	Interprofessional Competency-Based Assessment	Psychological Safety	Repeated and Distributed Practice	Attention to Differences and Hierarchy	Feedback during Debriefing	Sociological Fidelity	Program Evaluation	Train Facilitators	Institutional Support
**Program**	**1**	**2**	**3**	**4**	**5**	**6**	**7**	**8**	**9**	**10**	**11**	**12**
**A**	Partially Applied	**Fully Applied**	Partially Applied	*Not Applied*	Partially Applied	**Fully Applied**	Partially Applied	**Fully Applied**	Partially Applied	Partially Applied	**Fully Applied**	Partially Applied
**B**	**Fully Applied**	**Fully Applied**	**Fully Applied**	**Fully Applied**	Partially Applied	**Fully Applied**	Partially Applied	**Fully Applied**	**Fully Applied**	Partially Applied	*Not Applied*	Partially Applied
**C**	Partially Applied	**Fully Applied**	**Fully Applied**	*Not Applied*	**Fully Applied**	*Not Applied*	**Fully Applied**	**Fully Applied**	**Fully Applied**	Partially Applied	**Fully Applied**	Partially Applied
**D**	Partially Applied	Partially Applied	**Fully Applied**	*Not Applied*	**Fully Applied**	*Not Applied*	**Fully Applied**	**Fully Applied**	Partially Applied	*Not Applied*	*Not Applied*	Partially Applied
**E**^**a**^	**Fully Applied**	Partially Applied	**Fully Applied**	*Not Applied*	**Fully Applied**	*Not Applied*	Partially Applied	**Fully Applied**	**Fully Applied**	Partially Applied	Partially Applied	Partially Applied
**F**	*Not Applied*	**Fully Applied**	*Not Applied*	*Not Applied*	Partially Applied	**Fully Applied**	*Not Applied*	**Fully Applied**	Partially Applied	**Fully Applied**	Partially Applied	Partially Applied
**G**	Partially Applied	Partially Applied	Partially Applied	*Not Applied*	Partially Applied	Not Applied	*Not Applied*	Partially Applied	**Fully Applied**	**Fully Applied**	Partially Applied	Partially Applied

^a^Incomplete data with only 1 interview

Interviewees often emphasized the distinction between what they considered to be an ideal for IPSE, with the full application of the principles, and what they were able to do in their programs. An interviewee compared how facilitators in the program would implement “equitable distribution” in an ideal situation with what tended to happen during simulation sessions:

“So, in the ideal world, there’s co-facilitation between the physician and the nurse. I think every single session that I’ve been at, the physician typically takes, opens up the conversation. But, ideally, the nurse facilitators actually take on a big piece.” (Program A, Interview 1).

For programs B and F, we identified a lack of congruence between observation and interview data. In our observations, some principles were not fully applied (e.g., “psychological safety” for program B; and “equitable distribution”, “interprofessional competency-based learning objectives”, “attention to differences and hierarchy”, and “sociological fidelity” for program F). However, interviewees reported aiming to apply these principles in their programs.

### Affordances and barriers to IPSE

Data from interviews with program facilitators and developers helped us understand some of the affordances that supported sustainable IPSE programs as well as some of the barriers encountered. In addition, they helped us understand why some principles were more easily applicable than others in some programs. We describe these facilitators and barriers below.

### Interprofessional “Buy-in”: participant, facilitator, and institutional

Interviewees emphasized that getting people at all levels of training and from all professional backgrounds to believe in the value of the IPSE sessions was an important factor in the success of programs. This buy-in needed to come from participants, facilitators, and institutions. Buy-in had the potential to grow over time, as people’s experiences and interactions with the IPSE programs increased. Institutions’ buy-in led to the allotment of more resources, including money, space and time, thus increasing “institutional support”.

“Once [participants are] there…they’re very engaged…that wasn’t always the case. When we first started this out they would all sort of stand against the wall and be like, ‘I’m not doing anything, this is scary’ … But, that has changed. People have really started to realize, ‘this is important and I can learn something here and I want to participate.’” (Program A, Interview 1).

Interviewees discussed how choosing the right type and level of fidelity, or realism, was important to achieve participant buy-in. In particular, they noted that “sociological fidelity” – the extent to which the simulation mimics how people in real life interact [21] – leads to increasing buy-in from participants. Interviewees described attempting to achieve sociological fidelity by having everyone participate in their usual role, and developing scenarios that were similar to real patients that participants would encounter in clinical care. Interviewees also highlighted the importance of equipment fidelity in achieving buy-in, especially from participants. They noted that having simulation equipment that looks, feels, and responds in the same way it would in the clinical setting was important.

“If the monitor doesn’t look realistic people really lose their ability to understand what’s going on. So we work very hard to make sure that our monitors are in place of the actual patient monitor that would be there, that they look, in terms of color and sound, as realistic as possible, that the equipment that they use is all real equipment, kept in the right location…that adds to the learner’s perspective of realism in ways that are more meaningful.” (Program G, Interview 1).

### Resources: money, time and space

Interviewees cited resources such as money, time and physical space as important to the success of IPSE programs. In addition to increasing buy-in, these resources enabled the application of principles such as “program evaluation”, “interprofessional competency-based assessment,” and “repeated and distributed practice.”

“I think to do it more frequently would probably require additional support. For now, I think we are able to maintain what we have… I think there is interest from everyone, and most people who come say that we should do it more frequently. I think the challenges are to try to schedule a time that’s convenient for everyone.” (Program B, Interview 1).

“That funding kind of waxes and wanes, so right now we don’t have that much funding… So right now we’re in a little bit of a coasting phase where we’re just keeping the sims going, but we’re not really trying to improve the program or make any curricular changes. But we were able to do quite a bit of curriculum development and quality improvement within the simulation program. Previously over the last like two to three years where we had a half time patient safety coordinator who was really instrumental in that.” (Program C, Interview 1).

As these quotes show, more resources potentially enabled facilitators to run more sessions, thus allowing participants to attend more simulation sessions over time (“repeated and distributed practice”). More resources also enabled programs such as Program C to hire someone to evaluate teams during simulated sessions (“interprofessional competency-based assessment”) and to analyze the impact of the program (“program evaluation”) with the goal of improving the usefulness of the program. Yet, we found that most programs operated with limited resources and sometimes solely depended on the commitment of facilitators.

“It’s one of the things people find a lot of value in, at least by word of mouth. It was something that we definitely wanted to continue. The residents […], I think they find usefulness in it and we did too so we kept it on the schedule because it’s pretty important. […] If we weren’t to do it [run the program], I don’t know that anyone would put up much of a fuss but it’s something that people find a lot of value in. But we do have a lot of ownership of it to make it actually happen.” (Program D, Interview 2).

### Lack of outcome measures

In addition to limited resources, interviewees mentioned that their limited knowledge of instruments to assess team performance in simulation acted as a barrier to applying the principle of “interprofessional competency-based assessment.”

“I don’t think that we’ve had a good tool. And, then it’s also simply bandwidth; who’s going to do it, how do you record it, what do you do with the information, what’s really the purpose of the assessment? … I haven’t come up with a non-labor-intensive way to do it and we’re already so crunched right, for time… So, how do we fit it all in and then, what’s the cost benefit analysis of doing an assessment.” (Program A, Interview 1).

Furthermore, interviewees viewed the small-scale nature of their program as a barrier to undertaking “program evaluation.” All the programs involved in our study focused on a small number of professions at one department in a hospital, which limited the number of sessions and of participants per year.

“I don’t think there is enough N for that [evaluating the program], and then, additionally, to attribute it to a single course versus other medical center initiatives, I think, would be also difficult. I suppose you could look at codes that happened prior to 2010 and codes that have happened subsequently, after that, but we don’t have any of that information currently.” (Program B, Interview 1).

“[F]rom the standpoint of having the desired effect and again, our numbers aren’t big enough to measure impact but there’s other literature out there like from larger systems like in Massachusetts, they pulled three or four different birthing hospitals that started simulation programs and it did show an improvement in outcomes.” (Program C, Interview 1).

### Power discrepancies

The last major factor that we identified as influencing the application of principles in IPSE programs was power. Interviewees described how multiple forms of power discrepancies influenced the programs and sometimes prevented the full application of the principles “attention to differences and hierarchy,” “equitable distribution,” and “psychological safety”.

“I’ve never heard a nurse participant speak up and give feedback about the sim when a trauma attending is there or when trauma nursing leadership is there. The nurses are very quiet. I don’t hear interns asking questions. It seems a lot more constrained when there are high level administrators there.” (Program G, Interview 2).

Experience also created power discrepancies between simulation participants, preventing participants from sharing feedback to people whom they considered have more experience:

“And so I think it’s definitely awkward to critique one of your colleagues that’s an experienced ICU nurse as well. And most of them have more ICU experience than I do, or a couple of them do.” (Program B, Interview 2).

Interviewees also noted that power discrepancies between professional groups influenced interactions between facilitators and participants, as well as among participants. Some programs sought to mediate this by involving facilitators from multiple professions at each session (e.g., Program A had two physician and two nurse facilitators at each session), applying the principle of “equitable distribution” for both participants and facilitators. Other programs chose to only use facilitators from one professional group (e.g., Program C was facilitated by midwives).

“Physicians are not, in my experience, super well-suited to facilitate simulations because there’s an already imposed hierarchy that comes into play when a physician is running the code, and I feel like it does, to me, dampen that group participation when there’s a physician” (Program C, Interview 1).

## Discussion

Through this case study research of seven interprofessional simulation programs, we found variable implementation of 12 principles for effective IPSE we synthesized from the literature. Some principles such as “active learning”, “psychological safety,” “sociological fidelity”, and “feedback during debriefing” are commonly applied, while others such as “interprofessional competency-based assessment” or “repeated and distributed practice” are rarely applied. Based on the interviews with program facilitators, we believe that the full application of all 12 principles represents the ideal of IPSE, but important barriers prevented the programs we studied from accomplishing this ideal. In particular, we identified that buy-in, resources, lack of outcome measures, and power discrepancies influenced the extent to which principles were applied. The framework and results of our study can be used by those who plan a new ISPE program or want to optimize an existing program to consider the realities of implementation along with ideal features of IPSE programs.

 Reviewing the degree of application of the different principles, we noted that those grounded in SBE guidelines, such as “active learning”, “feedback during debriefing,” and “sociological fidelity” were more frequently applied. In contrast, those grounded in IPE guidelines, such as “interprofessional competency-based assessment”, “attention to differences and hierarchy,” and “equitable distribution” were inconsistently applied across programs, highlighting the difficulty of implementing IPE. Challenges to implementing IPE, such as limitations in resources, scheduling and stereotypes are well-known [22, 23]; thus, our data align with what is described in the IPE literature. When designing programs, special attention should be spent on these principles and known challenges. The four factors we identified as influencing the application of principles in IPSE programs – buy-in, resources, lack of outcome measures, and power discrepancies – are also notable in the literature surrounding the implementation of IPE. In the context of simulation, many of these factors may be accentuated because of the need for simulation space and equipment, as well as for facilitators who are not only adept in leading interprofessional sessions, but also ones that understand teaching and learning in the simulated setting. Despite these difficulties, we saw multiple programs continue to sustain themselves with limited resources, indicating a continued belief among program developers in the importance of IPSE in the training of healthcare professionals. We also saw the closing of one program during our study period. The director at Program E moved on to another job, and subsequently the program ended, highlighting the importance of institutional support, multiple leaders and distributed responsibility to keep a program running.

An additional challenge our study has uncovered is the limited use of evidenced-based outcome measures to evaluate IPSE programs. The limited application of “interprofessional competency-based assessment” and “program evaluation” in the programs we studied reflects the lack of clarity among program leadership as to what they are measuring, and how they should be measuring it. This sentiment is echoed in the literature, with multiple systematic reviews on IPSE assessments highlighting the lack of rigorously gathered validity evidence, including limited evidence that improvement in studied outcome measures leads to improved interprofessional collaboration and patient safety in practice [24, 25]. It also highlights the resource burden associated with assessment and evaluation. Recommendations to assess learners and evaluate programs will be met with resistance until further work is done to develop easy to administer measurement tools for teamwork and interprofessional collaboration, and resources are allotted within programs to conduct this important work. From our study, it is unclear whether program creators and facilitators consult the IPE literature and if they are familiar with the IPE competencies and the tools that already exist [26, 27]. Further work is needed to understand whether IPSE program creator and facilitators knowledge of IPE competencies is a barrier to its application in this setting.

In alignment with IPE literature, we found that power discrepancies between professional groups played a role in the implementation of IPSE programs [27–29]. Additionally, we found that power discrepancies based on status and experience affected how participants and facilitators engaged in IPSE, which has been observed in previous qualitative work on IPSE [8]. These findings highlight that individuals can draw on multiple sources of power, such as position in the hierarchy and experience [30]. These various bases of power need to be taken into account when designing curriculum aiming to improve interprofessional teamwork.

Our work highlights the gaps found between principles and practice in IPSE. In order to see the full effect of IPSE on interprofessional collaboration and patient safety, we must change our focus from the ideal of simulation as imagined to the realities of simulation as enacted. To this end we must not only consider the theory and design of IPSE programs, but also pay attention to implementation. Applying implementation science to in situ interprofessional simulation may decrease the gap between research and practice [9]. Implementation science is the “scientific study of methods to promote the systematic uptake of research findings and other evidence-based practices into routine practice, and hence, to improve the quality and effectiveness of health services” [31] and has been used in healthcare and educational settings.

Our study has important limitations. There was a two-year gap between the initial observations and interviews. While we asked interview questions that checked for program changes in the interim and found few, one program ended. A benefit of the delay was the opportunity to understand sustainability of programs and to highlight the role that program facilitators play in maintaining the programs. In addition, we did not discuss data from our observations with interviewees as we did not want interviewees to feel as though we were evaluating or critiquing their program. This limits our ability to explain discrepancies between observation and interview data for a few principles for programs B and F. We believe that these discrepancies are due to facilitators’ inability to apply principles as fully as they intend due to the barriers we identified in this study.

## Conclusions

 We found that IPSE programs varied in their implementation of IPSE principles derived from commonly used guidelines for IPE and SBE due to a number of important barriers. To truly change interprofessional teamwork, IPSE programs may benefit focusing on the how with implement programs to achieve the ideal. The gaps that exist between the ideals of IPSE and the realities of implementation may be narrowed by higher buy-in from simulation participants, facilitators, and institutions; by more resource allocation to IPSE programs; by development and sharing of instruments to assess learners and programs; and by acknowledgement power discrepancies and their impact on learning.

## Supplementary Information

**Additional file 1.**

## Data Availability

The datasets generated and/or analyzed during the current study are not publicly available due to individual participants privacy being compromised but are available from the corresponding author on reasonable request.

## Abbreviations

IPSE: Interprofessional Simulation Based Education
IPE: Interprofessional Education
SBE: Simulation Based Education
