# Supplementary material for: From ideal to real: a qualitative study of the implementation of in situ interprofessional simulation-based education
Source: BMC Med Educ. 2022 Apr 21;22:301. doi: 10.1186/s12909-022-03370-2 (PMC9027347; doi:10.1186/s12909-022-03370-2)
Supplement: Supplementary file 1 — Additional file 1. [file 12909_2022_3370_MOESM1_ESM.docx]

**IPSE Facilitator/Program Leader Interview Guide**

Guiding Research Question:

1. What does the structure and organization of current IPSE programs look like and to what extent do they follow or deviate from published recommendations?
2. What is the rationale behind current IPSE practices based on stakeholders’ perspectives?
3. What are the barriers programs face in development and implementation of IPSE programs and in aligning with published recommendations?

| IPSE Principle | Description |
| --- | --- |
| Equitable Distribution | Planning, implementation and learning should be done jointly at every level involving all relevant professions. There should be equal distribution of roles and responsibilities across professions. |
| Active Learning | Activities should promote learners’ cognitive engagement (active participants, not passive bystanders) using strategies such as multiple repetitions, feedback, task variation, or intentional task sequencing. |
| Competency Based Learning | Program should have clearly defined learning objectives that focus competencies for collaborative practice including interprofessional knowledge, behaviors/skills, and attitudes. |
| Psychological Safety | Programs should focus on setting up an atmosphere of social acceptance of feedback from all peers, through the physical environment and pre-briefing. |
| Repetitive and Distributive Practice | There should be an opportunity for learners to engage in focused, repetitive practice where the intent is skill improvement over a period of time (>1day of simulation). |
| Hierarchy | Educators need to be able to discern and address with sensitivity, diversity and differences between groups in educational, professional and cultural background, as well as issues related power, status and hierarchy among varying professions. |
| Feedback/Debriefing | Feedback, information on performance provided to the learner by the instructor, a peer, should be provided either during or after the simulation activity. Debriefing in IPE should be attributed to most experienced debriefers. |
| Sociological Fidelity | Scenarios should have high levels of social realism and reflect how teams in real life are arranged. (interprofessional teams are often ad-hoc in nature with changing membership). |
| Program Evaluation | Programs should be rigorously evaluated as early as possible and involve as many stakeholders as practical. The purpose of the evaluation should be stated clearly and consider learning outcomes and theoretical perspectives. Results may be considered for dissemination. |
| Train Facilitators | All IPSE educators should receive training to understand its ethos, principles and methods. Training should focus on how to develop, deliver and evaluate interprofessional simulation based education. |

IPSE published recommendations

**Interview Introduction Script**

I want to thank you for taking the time to meet with me today. My name is __________and I would like to talk to you about your experiences participating in the in situ simulation program at ___________. Specifically, we are trying to learn how current interprofessional simulation programs are working in practice, and we appreciate your expertise as a facilitator and/or program developer of ______ program. Specifically, we hope to understand how the history of your program, how it functions and its strengths and weaknesses. The interview should take less than an hour. I will be taping the session because I don’t want to miss any of your comments. Although I will be taking some notes during the session, I can’t possibly write fast enough to get it all down. Because we’re on tape, please be sure to speak up so that we don’t miss your comments. All responses will be kept confidential. This means that your interview responses will only be shared with research team members and we will ensure that any information we include in our report does not identify you as the respondent. Remember, you don’t have to talk about anything you don’t want to and you may end the interview at any time.

Are there any questions about what I have just explained?

Are you willing to participate in this interview?

**Date:**

**Program ID number:**

**Participant ID number:**

**Background info on simulation facilitator/program leader**

- What is your clinical job/role?
- How would you describe your role within the mock codeprogram? What are your responsibilities?
- Were you involved when the program was first designed/implemented?

**Program description & Design**

- When was your simulation program first implemented?
- Why was the program designed/implemented?
- What are the goals of the program?
  - Do you have these goals written down?
    - If yes, ask for a copy
  - Have these changed over time?
    - If yes,
      - How?
      - What prompted the changes?
  - Do you feel like the goals have been met?
    - If yes,
      - In what ways?
    - If no,
      - What are the barriers to achieving the goals?
      - What would it take to achieve the goals?
- Who was involved in designing the program? (check for multi-professional)
- Who provides the resources to run the program?
  - Who provides space?
  - How is time for staff nurses compensated?
  - How is time for trainees to participate provided?
  - How is time for facilitators compensated?

**Program Operation**

- Who runs the program?
  - What are their roles/responsibilities?
  - What was the reason for selecting these people in these roles?
- Who facilitates?
  - What are the roles and responsibilities of facilitators?
  - Are they the same people each time?
  - Do facilitators receive any training?
    - If yes,
      - What does the training involve?
    - If no,
      - Why do you think that is?
      - What are the barriers to doing so?
- Who participates (professional background, level/years of training)?
  - How frequently do participants participate?
  - Do participants have to complete a certain number or type of scenario?

**Session Structure**

Pre-Briefing

- Do you have a prebriefing?
  - If no,
    - Is this by design?
      - If yes,
        - Why?
      - If no,
        - Would you have a prebriefing in an ideal situation?
        - What are the barriers to having a prebriefing?
  - If yes,
    - what is the purpose of your pre-briefing?
    - Who (professional background) leads the prebriefing?
    - What do you discuss in your prebriefing?
    - Is this standardized?

Scenario Development

- Are your scenarios scripted prior to the session?
  - If yes,
    - How are scenarios created?
    - Who is involved in creating them?
  - If no,
    - Why not?
    - How are scenarios generated?
- Do you formulate learning objectives for your scenario?
  - If yes,
    - How are they developed?
    - Who is involved in the development of learning objectives?
  - If no,
    - Would you have learning objectives in an ideal situation?
    - What are the barriers to having learning objectives?
- Do you feel that each profession is well represented in the scenario?
  - If yes,
    - What do you perceive that your program does to achieve this?
  - If no,
    - why do you think that is?
    - what are the barriers to doing so?
- Do participants take on roles similar to their clinical roles in real life?
  - If no,
    - How are their roles different?
    - Why do they play different roles?
- Do participants generally behave in ways similar to how they do in real life? (specifically can ask about interprofessional interactions if does not come up)
  - If no,
    - How do they act differently?
    - What do you think prevents them from acting how they normally would?

Debriefing

- Does your program have a debrief?
- Is there a structured format? Please explain
- What do you see as the purpose of the debrief?
  - What is generally discussed during debrief?
    - Are the topics of power, hierarchy, interprofessional bias discussed?
      - If yes,
        - Do you feel comfortable and prepared to discuss these topics?
- How would you characterize a successful debrief?
- Who (professional background) facilitates the debrief?
- Do you feel that all participants feel comfortable talking and contributing to the discussion during the debriefing?
  - If yes,
    - why do you think that is?
  - If no,
    - what are the barriers to doing so?

**Evaluation and Assessment**

- Are participants assessed in any way? (formally or informally)
  - How are they assessed?
  - What is the purpose of the assessment?
- Do you evaluate your program?
  - If yes,
    - How do you evaluate your program?
    - How frequently?
    - What is the purpose of the evaluation?
    - Who evaluates the program?
  - If no,
    - Why do you think that is?
    - What are the barriers to evaluating the program?
